# Exploring the Impact of Learning Activities Supported by 360-Degree Video Technology on Language Learning, Intercultural Communicative Competence Development, and Knowledge Sharing

**DOI:** 10.3389/fpsyg.2021.766924

**Published:** 2021-11-26

**Authors:** Rustam Shadiev, Jiatian Yu, Wayan Sintawati

**Affiliations:** School of Education Science, Nanjing Normal University, Nanjing, China

**Keywords:** 360-degree video technology, knowledge sharing, language learning, intercultural communicative competence (ICC), perceptions of technology-assisted learning

## Abstract

This study integrated intercultural learning activities into English as a foreign language (EFL) learning course in a vocational school in China. The study focused on improvement of students’ EFL abilities, intercultural communicative competence (ICC), and knowledge sharing (KS). A group of second-year students from China were partnered with a group of university students from Indonesia. 360-degree video technology was used to create an authentic and immersive intercultural learning environment in which students created content in English related to their culture and traditions, shared content with partners from the other culture, and reflected on their intercultural learning. We investigated whether learning activities supported by 360-degree video technology have positive impact on EFL learning, ICC development, and KS. The data was collected through questionnaires, tests, observations, and interviews. Three main findings were obtained in the study. The results demonstrated that 360-degree video technology-supported intercultural learning activities improved students’ EFL abilities, ICC, and KS. In addition, it was found that dimensions of KS and ICC have significant relationship with each other. Finally, the students had a positive attitude toward the learning activities supported by 360-degree video technology, were satisfied with the technology, and had intentions to use it in the future for learning. On this basis, we made several suggestions for educators and researchers.

## Introduction

In the course of language teaching, the acceptance of culture as a key dimension has a significant place (Atay et al., 2009). The increasing intercultural connections in our globalized society require language teachers to cultivate the skills of interculturality and integrate their development into their instructional goals (O’Dowd, 2016; Çiftçi and Savaş, 2018; Üzüm et al., 2020). The main purpose of foreign language teaching has been identified by many researchers as developing the intercultural communicative competence (ICC) of foreign language learners (Byram, 1997; Byram et al., 2002; Atay et al., 2009; Rissanen et al., 2016). Therefore, this research integrated intercultural learning activities into formal school classrooms. Through cultural exchanges with foreign language learners from the target culture, students applied foreign languages to real cultural exchanges to enhance their ICC and language proficiency.

The term intercultural refers to the communication of two cultures or languages within or out of the boundaries of nation-states (Kramsch, 1998). In the area of language pedagogy, ICC was introduced to complement the concept of communicative language teaching (Kusumaningputri and Widodo, 2018). In fact, by improving ICC, students can acquire multidimensional skills. For example, such skills may include knowledge, attitudes, skills of interpreting and relating, skills of discovery, and interaction and critical cultural awareness (Byram, 1997).

Existing literature showed that technology-supported intercultural and language learning have many advantages. According to Sevilla-Pavón and Haba-Osca (2017), technology-supported language and intercultural learning beneficial in terms of the development of different skills and competences (namely linguistic, digital, and intercultural) and motivation at the same time. Oakley et al. (2018) also claimed that the technology-supported language and intercultural learning were successful to a degree in supporting students’ learning in the areas of language, intercultural understanding and twenty-first-century skills, including digital literacies, and technological skills. Besides, students’ intercultural understanding conception moved from understanding to acceptance and respect (Wang, 2020). And Mitchell (2018) suggested that technology can be advantageous to achieve a personal connection with the target culture(s) inside the foreign language classroom and subsequently develop learners’ intercultural awareness. Furthermore, language learners can experience various cultures and enhance their perceptions toward language learning through meaningful learning situations relevant to real-life communicative events (Jung et al., 2019). In previous studies, it was found that participation and communication from two different cultures and above was the main way to improve ICC. This was the main method used in the study’s activities.

At the same time, in the field of language learning, researchers postulate that navigating in virtual worlds can create new experiences conducive to learning languages (Lan, 2014). With the use of virtual reality (VR) technology, users are totally immersed in the environment, and their experiences are closer to the real world. This opens the door to creating intercultural encounters in virtual environments (Liaw, 2019). Some studies have shown that virtual learning environments can assist the acquisition of cultural knowledge and intercultural communication competence, as they allow high-fidelity simulation of new cultural settings and bring realism to simulated cultural interactions (Allwood and Schroeder, 2000; Hasler and Friedman, 2012). Choosing 360-degree video technology to support language learning and intercultural learning is also complementary to research in related fields. At present, most of the relevant research uses computer-simulated VR scenes. This study used 360-degree video technology to allow students to personally shoot real scenes for intercultural peer viewing to bridge the gap; characters in typical VR learning environments are typically depicted in an unrealistic cartoonish style and physical environments lack detail. Virtual environments, both purposefully built for learning purposes and applications of the technologies from the digital wild, have been found to be beneficial to learning an additional language and intercultural communication. They have helped students to acquire L2 vocabulary (Sundqvist, 2019), improve speaking performance (Lan, 2014), achieve better essay writing scores (Lan et al., 2019), and enhance understandings of different ways of communication with people of diverse backgrounds. One important contributing factor is the social affordances provided by the technology as immersive environments offer possibilities for interactions and language socialization (Sykes et al., 2008). However, 360-degree video technology – a type of VR technology was rarely applied to foreign language classroom research. This research was a new attempt.

In this study, we introduced intercultural learning activities supported by 360-degree video technology for students from China and Indonesia. Their language learning skills, ICC and KS abilities were measured to explore whether learning activities supported by 360-degree video technology can facilitate these variables.

At the same time, this research had innovations. This study intended to use 360-degree video to allow students to personally shoot real scenes for intercultural peer viewing to bridge the gap that characters in VR learning environments were typically depicted in an unrealistic cartoonish style, and that the physical environments lack details. Moreover, because students were not native-speakers and had accent, this could bring some problems in communication and misunderstandings. We used subtitles in English in 360-degree videos to help comprehension of viewing content. This is one innovative solution that we have not seen in any related studies on 360-degree video technology applications to education. Finally, although some studies have focused on knowledge sharing (KS) in language learning, to the best of our knowledge, there no studies on KS in the area of intercultural learning. In the process of intercultural communication or interaction, KS often occurs and should not be ignored. At the same time, 360-degree video technology-assisted learning activity design and ICC assessment based on the Byram’s model in this study can also provide reference value for subsequent intercultural communication activities.

## Literature Review

### Intercultural Learning Supported by Technology

The interrelationship between language and culture is the core of language learning (Chen and Yang, 2016; Shadiev et al., 2018). Since the 1980s, intercultural language teaching methods have aimed to train students who are “intercultural speakers,” i.e., those who can explore cultural differences, analyze and interpret cultures, and avoid stereotypes and prejudices (Byram et al., 2002). According to Council of Europe and European Commission (2000), intercultural learning refers to the process by which individuals acquire knowledge and change attitudes or behaviors in the interaction of different cultures. Intercultural learning interprets the peaceful coexistence of people with different backgrounds, emphasizing understanding oneself, then understanding others, and understanding the differences between them. Cunha and Gomes (2009) regarded intercultural learning as an educational method to ensure intercultural dialog. They believe that intercultural learning is a social education process that promotes positive relationships between people and groups of different cultural backgrounds, and its purpose is to change ethnocentrism, resist prejudice, promote unity, and support human equality, dignity, and respect for diverse cultural identities. Directly communicating with partners from other cultures is the best way to achieve intercultural learning, but due to geographic location, this can consume considerable time and money.

In the current research, many technologies and tools have been proven to help learners communicate more conveniently with people from target cultures, such as video conferencing, instant messaging technology, and social networks. For example, Kohn and Hoffstaedter (2017) focused on nonnative English and German lingua franca conversations between pairs of students from schools in France, Germany, Netherlands and Spain. Learning activity support included the BigBlueButton video communication platform and Moodle Chat. Kohn and Hoffstaedter (2017) found that ICC development was significantly improved, and transcending the foreign language classroom to include elements of real life became a tangible experience for the students and teachers involved. Mitchell (2018) explored the use of Pinterest to encourage learners’ intercultural awareness and found the visual nature of Pinterest provided an inside view into the target culture and allowed learners to connect on a more personal level with the culture and subsequently develop their intercultural awareness. Lomicka and Ducate (2021) encouraged participants to complete tasks based on the four areas of social pedagogies and then to be more aware of their surroundings and document their linguistic landscape visually and in digital form. The researchers found that participants used observations in tasks to shape their own ideas about the target culture, and participants gained skills in intercultural learning as they reevaluated both their own and the target cultures while considering new perspectives as well as historical and cultural contexts.

In previous studies, most technologies that supported intercultural and language learning were mobile phones or computers connected to the Internet. These are now commonly used tools for students and provide text and two-dimensional images, movies, and animations. Such media lacks realism so that students have less interest in them. Researchers need to provide participants with technologies that can create authentic learning environments in which students can be fully immersed to add more interest and realism to students’ intercultural learning process. One such potential technology is 360-degree video technology.

### 360-Degree Video Technology

The 360-degree video is also called an immersive spherical video (Hosseini and Swaminathan, 2016). Creating immersive spherical video generally costs less than creating an animation-based VR (Sung et al., 2018). In addition, 360-degree video technology enables users to view a 360-degree picture or video in which objects, situations or scenarios look real, so the realism in such environments is also high (Ye et al., 2019). Users can use head-mounted displays, such as Oculus VR glass, HTC VIVE VR glass or MI VR glass, to watch 360-degree pictures or videos.

Since 360-degree video is immersive content, it allows viewers to browse around so that they can choose and control what they view (Walshe and Driver, 2019). Therefore, it provides participants with multisensory modalities, including visual, auditory, and kinesthetic modalities, to experience the event, thereby ensuring the true performance and presentation of the environment in the virtual environment (Barić et al., 2020). Because of such advantages, this technology has been used in many educational studies. Smith and Townsend (2020) applied 360-degree video technology to the scientific content-based EFL courses of Japanese universities and found that the 360-degree project provides an effective forum to integrate content language and concepts and effective English expressions into the classroom. In the study of Walshe and Driver (2019), scholars explored how the use of 360-degree video can support student and teacher reflection in a university, and the results suggest that the immersive, embodied experience of reflecting using 360-degree video develops a more nuanced understanding of microteaching practice and supports student teachers’ teaching self-efficacy. Shadiev et al. (2020) designed intercultural learning activities supported by 360-degree video technology for students from universities in China and Uzbekistan and found that in the process of intercultural learning, participants gained a sense of presence, immersion, and authentic cultural experience. Participants were satisfied with related learning activities, and their intercultural competence improved.

From previous research, it can be found that 360-degree videos can integrate more content, include more details, support teachers’ and students’ self-efficacy, and provide a sense of presence, immersion, and real experience. However, most studies have focused on 360-degree video content created by the instructors which is contradicts student-centered learning principles. Furthermore, little attention has been given to intercultural learning in formal foreign language classrooms using 360-degree video technology.

### Intercultural Communicative Competence

Scholars have different concepts about ICC. ICC is the expansion of communication skills and the ability to be displayed when communicating with people from other cultural and language backgrounds. Mirzaei and Forouzandeh (2013) conceptualized ICC as the communicative ability to understand and negotiate both linguistic and cultural differences with people of other cultures appropriately using language as well as the capacity to relate to otherness effectively. Considering economic globalization, the importance of intercultural learning in foreign language classrooms is self-evident. Given the role of English as a global lingua franca, ICC has been the pillar of ELF or English as an additional language instruction inasmuch as speakers of English have different linguistic and sociocultural backgrounds (Galloway, 2017).

In recent years, many studies on foreign language learning have focused on ICC. Lee and Song (2019) compared foreign language learners’ perceived ICC development under three different conditions over 6 weeks: (1) a study-abroad program in Korea, China, Japan, France, and Spain designed for American undergraduates; (2) telecollaboration between Korean learners of English and American learners of Korean; and (3) an on-campus language study among Korean learners of English and American learners of Chinese who were learning languages at their home institutions. Scholars have argued that online interactions with members of the target culture can be as beneficial as studying abroad and that it is at least more beneficial than traditional classroom language learning in the development of learners’ perceived ICC. Ryshina-Pankova (2018) examined written synchronous chats created throughout a 7-week tele-collaborative activity by advanced American learners of German at a private United States university and by German university students. Ryshina-Pankova (2018) found that the different strategies contributed to a greater or lesser display of ICC and at the same time enabled different opportunities for further development of the latter. Liaw (2019) investigated the effects of using open social VR for university English as a foreign language (EFL) on learners’ ICC and claimed that occurrences of ICC were identified in the participants’ interactions with international interlocutors.

### Knowledge Sharing

It was found that participation and communication from two different cultures are the main ways to improve ICC. As an important dimension of ICC, knowledge is of great research significance. Communication is the process of KS. KS can make knowledge creation of greater value (Woodfield and Husted, 2017). KS between partners can achieve intercultural knowledge development and further intercultural attitudes, skills, and awareness. This research will explore KS from three dimensions: organizational communication, personal interaction and practice.

Organizational communication includes behaviors of sharing knowledge in formal interactions within or across teams or work units (Yi, 2009). For example, a learning group may hold discussions in class to brainstorm ideas or solve problems by asking group members for their opinions. This means that group members believe that through KS, they can help the entire organization achieve their goals rather than being out of self-interest (Gurteen, 1999). Members may contribute because they think that their contributions are valuable to the organization, and contributing provides positive social feelings or promotes a sense of personal responsibility (Cabrera and Cabrera, 2002). This type of KS emphasizes the sharing of default knowledge through formal face-to-face dialog.

Personal interaction includes behaviors of sharing knowledge in informal interactions among individuals, such as chatting over lunch and helping other members who approach them (Yi, 2009). The intrinsic motivation of this type of KS is high because sharers perceive KS as self-determined (Kaser and Miles, 2001). Obviously, the better an individual’s interpersonal relationship is, the greater the opportunity for an individual to share knowledge with people they know (Kubo et al., 2001).

Practice means that knowledge is shared through informal social interactions of a person-to-group channel (Yi, 2009). KS of this dimension takes place when group members communicate in an informal and personal way around topics of common interest. Kaser and Miles (2001) called this behavior social exchange relationship-based behavior (a social exchange relationship is reciprocal acts in which individuals offer help to one another).

Previous studies on ICC lacked the exploration of knowledge and KS, which is the gap that this study wants to make up for. At the same time, the exploration of ICC has been ongoing. Applying different technologies, focusing on different stages, and conducting research may provide references for the subsequent teaching activities to improve students’ ICC in foreign language classrooms.

### Research Motivation and Research Questions

Language ability, ICC, and KS skills development is the main target in most foreign and/or second language learning curricula. Language instructors and researchers apply various technologies to assist teaching and learning process. However, most of existing technologies (e.g., email, discussion boards, instant messaging, or social networks) were not capable to create such learning environments in which leaners could have authentic, immersive and sense of presence learning experiences. To address this issue, we applied 360-degree video technology that supported intercultural virtual exchange among students from two countries. Furthermore, in contrast to other research on 360-degree video technology, learning process was based on students-centered approach as students in this study created culture-related content by themselves and shared it with their foreign partners. Our focus was on language ability, ICC, and KS skills development which is different from that of other related studies on 360-degree video technology in education. Therefore, the value of this study for the literature and its contribution to theoretical knowledge is that it creates intercultural learning environments for virtual exchange based on 360-degree video technology and presents evidence suggesting that the created learning environments can facilitate language ability, ICC, and KS skills.

In this study, we aimed to address the following research questions: (1) What is the impact of intercultural learning activities supported by 360-degree video technology on students’ ICC? (2) What is the impact of intercultural learning activities supported by 360-degree video technology on students’ KS? (3) How do the participants perceive the usefulness of learning activities supported by 360-degree video technology? What is the impact of intercultural learning activities supported by 360-degree video technology on students’ language ability?

## Materials and Methods

### Participants

The participants in this study were from China and Indonesia. Chinese participants were 35 second-year students from a vocational school majoring in tourism (12 boys and 23 girls). They were between 16 and 17 years old. Indonesian participants were recruited using convenience sampling from local colleges based on their availability and willingness to participate. Five Indonesians participated in this study (3 men and 2 women). They were between 18 and 23 years old, majoring in mathematics, biology, geophysics, cooking, or agricultural engineering.

At the time of the study, the participants from China were vocational school students. To entered the vocational school, they have completed elementary (6 years) secondary (3 years) schools. After finishing the vocational school (3 years), they are able to enter a college. Their English proficiency has reached the intermediate level. They have mastered certain vocabulary, grammar, and dialogue knowledge, and could carry out basic dialogues. After some preparation, they could also carry out complex interactions. Indonesian students were university students. They also completed compulsory elementary and secondary school before entering the university. Their English proficiency has reached the intermediate level or above. They have mastered more vocabulary, grammar, and dialogue knowledge, and could carry out basic dialogues. After some preparation, they could also carry out complex interactions. Participants in China and Indonesia had a foundation in English, and their EFL level ranged from intermediate to advanced levels. The Chinese and Indonesian students in this study have experienced a complete basic EFL education, and they needed to develop their communication skills and competence, and this study provided such opportunity.

The ethics issues and approvals under which the data were collected and reported were considered in this research. All participants were informed about this research (i.e., its aims, activities, duration, data collection, etc.) before their participation. All of them agreed to participate in the research. The participants had no prior experience using 360-degree video technology, and this was the first time they participated in any intercultural learning activities. Therefore, all of them received detailed on-site guidance and hands-on practice to use 360-degree video technology prior to learning activities. In addition, instructional materials regarding learning activities and operating the 360-degree video technology were distributed to participants.

### Study’s Context

Two countries, China and Indonesia have been selected as the context where the study has been conducted because of EFL policy in these countries and availability of participants. English is the main foreign language in China and Indonesia. In both countries, learners learn EFL from very young age. Evidence shows that China is first and Indonesia is second in terms of the number of young EFL learners in elementary schools (Zein, 2017). The school system in both countries is similar. That is, both countries have a 6–3 school system, i.e., 6 years of primary school and 3 years of junior high school. Indonesia has included EFL as a compulsory subject from the first grade of elementary school whereas China has included EFL as a compulsory subject from the third grade of elementary school. However, most schools in China offer EFL classes from the first grade. It can be seen that both countries can attach importance to and guarantee basic education of EFL. Although the current language policy of Indonesia ensures that the language is taught to children from elementary and junior high school level. As a reflection of a language that has not been prioritized in school curriculum, school leavers largely have limited grasp of the language by the time they enroll into university programs (Lotfie, 2018). The same problem also appears in China, especially in the vocational education that some students enter after junior high school. Because at this stage, the school pays more attention to professional education.

As two countries have good relationships with each other, many projects were initiated between them in the field of education, business, politics, health, etc. For example, China and Indonesia signed an agreement to strengthen their educational partnership in 2017. As a result of partnership, many Chinese language centers have been established in Indonesia. In addition, there are many students from Indonesia who study in China for different degrees (e.g., bachelor, master, or doctoral) under variety of scholarships provided by the government of China. One researcher of the study was Indonesian who received her master of science degree from the university in China and she located in Indonesia at the time when this study was carried out. We asked her to help the study (e.g., to find participants in Indonesia, to instruct and guide them during the learning activity, and collect research data) because she is experienced in academic and research field and she kindly agreed. Participants in China were selected based on a convenience sampling method. They were from the school with which the other two researchers of the study collaborate for several years. The researcher selected a class in which the instructor and students were most willing to participate in the study.

### Learning Course

Intercultural learning activities were embedded into the compulsory English course for Chinese students arranged by the school. The students learned the language using a textbook. The teacher of the course was an experienced English teacher. She combined the content of the textbook with intercultural learning activities that her students had interest in and around the following topics: music culture, drink culture, food culture, costume culture, and family rules and members. Intercultural learning activities were extracurricular activities for Indonesian students.

The learning activities lasted for 8 weeks, with two 40-min sessions per week. In the beginning, the teacher illustrated and explained the learning procedure and overview of Indonesia and its culture to Chinese learners. To help students consolidate the learning content, at the end of each lesson or each task, the teacher randomly asked questions or encouraged students to share their feelings and experience about content with everyone. Then the instructor gave some comments or suggestions. Chinese students mainly completed script about they are going to present, group discussion of scripts and ideas sharing in class. After scripts were finalized, all of them completed 360-degree video shooting after class. Meanwhile, the researcher in Indonesia explained the learning process to the Indonesian participants. Then, Chinese students were divided into small groups of seven or eight students. Each group selected one topic of interest. The five Indonesian participants also selected one topic each. Chinese students and Indonesian participants who chose the same topic were matched to become communication partners. Therefore, Indonesian participants and Chinese students formed a one-to-many exchange partnership. In previous studies, Menard-Warwick et al. (2013) and Cunningham (2019) have also established a one-to-many communication partnership in intercultural research. Then, exchange activities were implemented. Participants completed the following core tasks in order.

1. Making introductions. Participants needed to introduce themselves and record them with a 360-degree video camera. Introductions included the following information: self-introduction; the reason why they choose this topic; introducing the culture such as its origin, development, representative artifacts, and its role in society. Chinese students were encouraged to share knowledge related to the target culture and to discuss their ideas for video content in groups.
2. Watching introduction video and questioning. Participants (Chinese and Indonesian) were asked to watch a video created by the foreign partner(s) using VR glasses. They were encouraged to ask questions related to cultural topics, such as the differences between the two cultures or what they were interested in. Chinese students were also encouraged to have group discussions after watching the videos and to pose questions to their Indonesian partner.
3. Responding to questions. Participants (Chinese and Indonesian) had to answer questions raised by foreign partners and record their responses using a 360-degree video camera. Chinese students were encouraged to have KS and group discussion to prepare their answers to questions of a partner.
4. Watching response video. Participants watched responses from foreign partners using VR glasses. Participants had to derive answers to their questions from the video.

On average, each task took about 2 weeks. Chinese students prepared scripts for shooting a video, discussed scripts’ content and shared ideas in groups and then recorded 360-degree videos. Each video lasted between two to 6 min. The communication between the participants was asynchronous and was carried out in English.

Indonesian students shot their videos first. Chinese students then watched partners’ videos and then shot their own videos. In videos, students asked each other questions related to cultural topics, e.g., what is typical traditional building in your city? Then students recorded their videos to answer such questions.

### 360-Degree Video Technology

**FIGURE 1:**
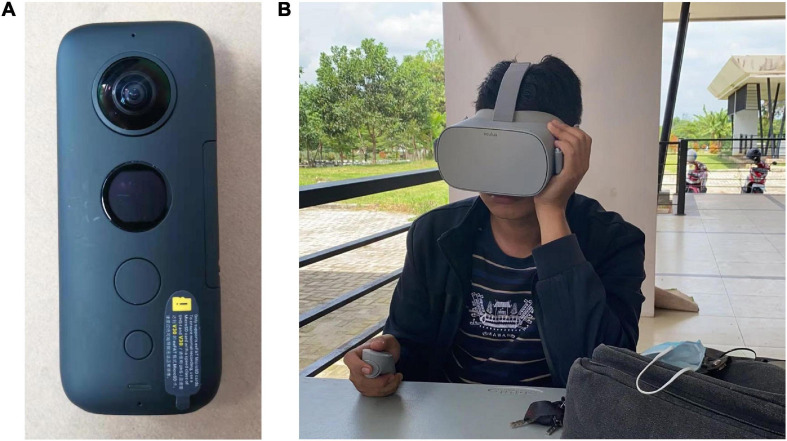
**(A)** Insta 360 one (https://www.insta360.com/) and **(B)** MI VR glasses (https://xiaomi-mi.com/). Image source: Author’s own images.

**FIGURE 2:**
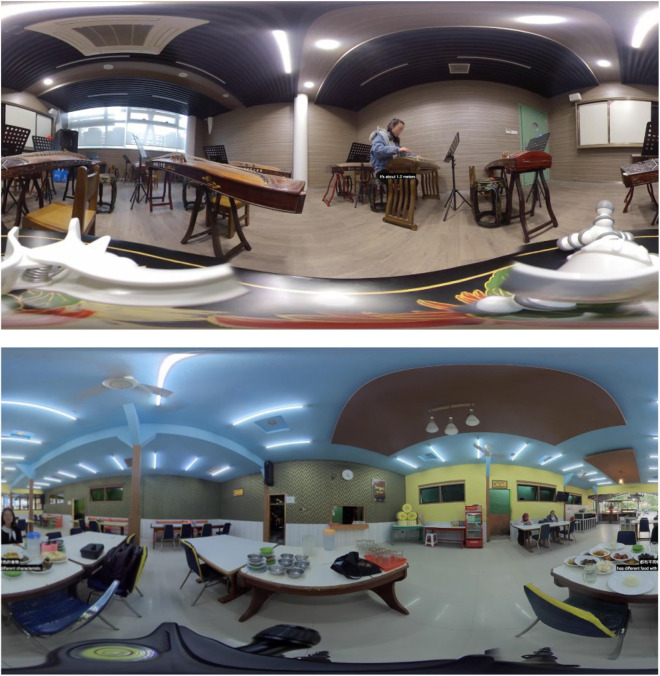
Screenshots of 360-degree videos.

360-degree video technology used by the participants includes a camera to record video (i.e., Insta 360 one x/r) and VR glasses (i.e., MI and Oculus VR glasses). Insta 360 one x/r (shown in Figure 1) can independently capture a 360-degree panorama of the user’s surroundings in the form of video. Figure 2 demonstrates two screenshots of 360-degree video: (a) a Chinese student introduces local musical instruments and (b) an Indonesian student introduces local food. MI (shown in Figure 1) and Oculus VR glasses are head-mounted all-in-one machines, which can be used without attaching a mobile phone to it. A total of 360-degree videos were shared among participants through the Google Drive platform.

It is worth mentioning that Insta provides a series of technologies for editing 360-degree videos. Researchers used these techniques to add subtitles in English and Chinese to videos taken by participants. The researcher used the Insta plug-in to add subtitles in Adobe Premiere CC 2020. Because the participants from China and Indonesia were nonnative English speakers, their speech in English had accents or errors. To avoid any misunderstandings, subtitles were included in videos, and they could help participants better understand the content of the video.

### Research Procedure

**FIGURE 3:**
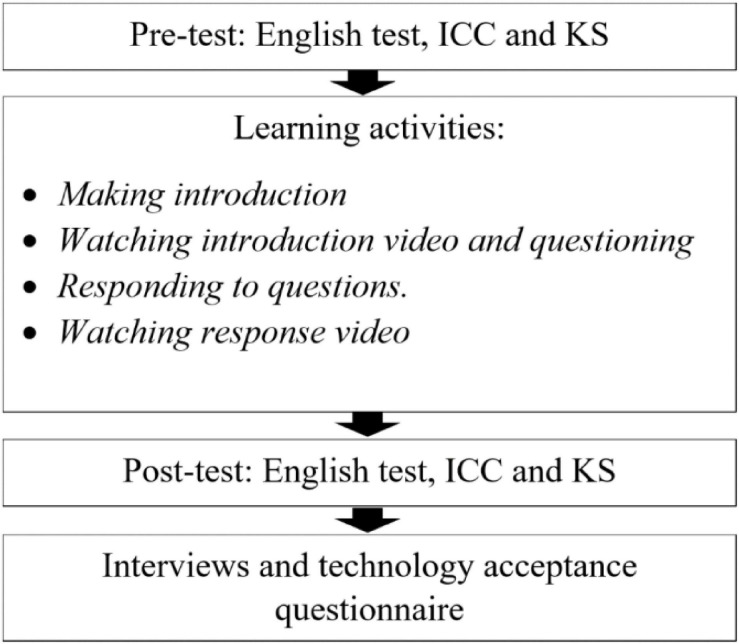
Research procedure.

The research procedure is shown in Figure 3. We used paper-based questionnaires to assess the ICC and KS of the Chinese participants before the learning activity (pre-test). Then, the participants were informed about the study. After that, the participants participated in learning activities using 360-degree video technology. When learning activities ended, we used another paper-based questionnaire to survey the participants’ ICC and KS (post-test). Participants’ views on VR technology were also examined. The English proficiency of Chinese students was collected through examinations organized by the school before the school semester and after it.

In addition, a few participants from the two countries were selected, and the researchers interviewed them. Three researchers (not teachers) assisted students in the two countries in the learning activities and collected and analyzed research data.

### Data Collection

To address three research questions, quantitative and qualitative data were collected. Items of the *Intercultural Communicative Competence Questionnaire (ICCQ)* was adopted from Fantini (2000). The questionnaire was based on Byram’s ICC model and included five dimensions with 40 items: knowledge (10 items), attitudes (10 items), skills of interpreting and relating (3 items), skills of discovery and interaction (7 items), and critical cultural awareness (10 items). These items were rated on a 5-point Likert scale with endpoints of completely disagree (1) and completely agree (5). The scope of intercultural competence was narrowed to student competence related to the topics covered in this study. The assessment scope of the participants’ ICC was only for the cultural topics involved in this study.

The *Knowledge Sharing Scale* was used to measure KS among Chinese participants. The scale was adopted from Yi (2009) and included three dimensions with a total of 22 items: organizational communications (8 items), personal interactions (8 items), and practice (6 items). These items were rated on a five-point Likert scale with endpoints of completely disagree (1) and completely agree (5).

The internal consistency of each questionnaire was checked by Cronbach’s alpha. The values in each dimension ranged from 0.832 to 0.955, which demonstrated satisfactory reliability.

The *Usefulness of Technology Questionnaire* was used to survey participants’ views on the use of 360-degree video technology in intercultural learning activities. It was adopted from Liaw (2019) and included three dimensions with a total of 18 items: learning activities (6 items), perceived satisfaction (5 items), and perceived usefulness (7 items). These items were rated on a five-point Likert scale with endpoints of completely disagree (1) and completely agree (5).

All three research instruments (i.e., the ICCQ, knowledge sharing scale, and usefulness of technology questionnaire) were widely used in educational research. Scholars argued that these research instruments are valid and reliably measures targeted research variables (Fantini, 2000; Yi, 2009; Liaw, 2019). In addition, we employed these instruments in our earlier related studies (see Shadiev and Huang, 2016; Shadiev et al., 2019, 2020, 2021), and we found that these research instruments are appropriate for school and university students and can be used in technology-assisted intercultural learning context effectively.

#### One-on-One Interviews

Each interview lasted for more than 20 min. The participants were asked questions about their participation in learning activities and their learning experiences. Some of the questions were as follows: What activities did you complete in this project? Do you think the project has improved your ICC? Give an example. Do you think the project has improved your KS? Give an example. What do you think of the VR learning experience? What problems did you encounter when using VR? The content of the interviews was transcribed and then analyzed.

#### Field Notes and Video Content

The researchers observed the learning behavior and interactions among students and took notes. The researchers also transcribed video content taken by the participants and analyzed it. Such data were used to support conclusions drawn from the quantitative data.

The *English Proficiency Test* was organized by the school at the beginning and end of the semester. The English test was divided into subjective and objective questions, including phonetic questions (5′), communicative dialog (5′), multiple choice (20′), cloze (10′), reading comprehension (15′), choice of words to fill in the blanks (10′), and translation and writing (35′).

## Results

### Participants’ Intercultural Communicative Competence Development

The results of the paired sample *t*-test showed that there was a significant difference between the ICC pre- and post-test in five dimensions. The results suggest that the participants developed ICC in all five dimensions. This means that students have improved intercultural knowledge, attitudes, skills of discovery and interaction, skills of interpreting and relating and critical cultural awareness. In the following parts, we discuss our results in details.

#### Knowledge

The participants scored significantly higher on the post-test (total *M* = 3.95; *SD* = 0.279) than on the pre-test (total *M* = 2.98; *SD* = 0.378), *d* = 1.73, *p* < 0.05 (Table 1). The results show that the participants’ intercultural knowledge increased. This demonstrates that intercultural activities improved participants’ understanding of the foreign cultures they come into contact with.

**TABLE 1:** Knowledge dimension of ICC.

**No.**	**Items**		**Mean**	***SD***	***t***	***p***
1	I know what intercultural communication is.	Pre-test	3.40	0.914	−3.431	0.002
		Post-test	4.17	0.857		
2	I understand the Chinese lifestyle and values.	Pre-test	3.46	1.094	−4.047	0.000
		Post-test	4.37	0.598		
3	I understand Chinese social etiquette.	Pre-test	2.91	0.781	−7.285	0.000
		Post-test	4.29	0.667		
4	I understand the basic knowledge of intercultural communicative activities.	Pre-test	3.26	0.919	−4.170	0.000
		Post-test	4.11	0.718		
5	I understand Indonesian values and lifestyle.	Pre-test	2.63	1.165	−5.464	0.000
		Post-test	3.86	0.845		
6	I know the social rules in Indonesia.	Pre-test	2.66	1.235	−4.203	0.000
		Post-test	3.60	1.006		
7	I understand what aspects of privacy are for people from other cultures.	Pre-test	3.14	1.033	−2.580	0.014
		Post-test	3.69	0.993		
8	I understand Indonesian customs, culture, daily activities of students.	Pre-test	2.54	1.120	−4.917	0.000
		Post-test	3.69	0.963		
9	I understand the mode of interpersonal communication in intercultural communication activities.	Pre-test	3.34	0.938	−3.894	0.000
		Post-test	4.00	0.804		
10	I understand basic information about Indonesia (geographical location, historical story).	Pre-test	2.51	1.067	−4.866	0.000
		Post-test	3.69	0.993		
	*Total*	Pre-test	2.98	0.378	−7.379	0.000
		Post-test	3.95	0.279		

The participants mentioned that they had almost no knowledge of the foreign culture before participating in this activity, so they did not know the basic knowledge or customs of the foreign culture and only had some etiquette of interpersonal communication. In addition, the participants said that before the learning activity, they could not state the similarities and differences between Chinese and Indonesian cultures, nor could they give specific impressions of the target culture.

Participants also had never thought of having direct or indirect exchanges with foreign students in the school curriculum. Participants gained information about foreign cultures, values and traditions on certain topics by participating in the learning activity. All participants stated that they had acquired knowledge about culture, customs, and living environment. The results suggest that intercultural learning activities supported by VR have greatly benefited students.

#### Attitudes

The participants scored significantly higher on the post-test (total *M* = 4.20; *SD* = 0.145) than on the pre-test (total *M* = 3.65; *SD* = 0.082), *d* = 1.57, *p* < 0.05 (Table 2). The results showed that the participants’ intercultural attitudes improved significantly. This demonstrates that intercultural learning activities affected participants’ attitudes toward foreign cultures. The participants said that they had never communicated with foreigners during their years of learning foreign languages and had no experience in intercultural learning.

**TABLE 2:** Attitude dimension of ICC.

**No.**	**Items**		**Mean**	***SD***	***t***	***P***
1	I am willing to communicate with people from different cultures.	Pre-test	3.60	0.914	−3.751	0.001
		Post-test	4.31	0.900		
2	I am willing to work hard to improve intercultural communication skills.	Pre-test	3.76	0.944	−2.001	0.043
		Post-test	4.29	0.825		
3	I am willing to understand the characteristics of intercultural learning and communication activities.	Pre-test	3.74	0.919	−2.451	0.020
		Post-test	4.23	0.731		
4	I will not give up when I encounter setbacks in communicating with people from different cultures.	Pre-test	3.57	0.917	−2.119	0.041
		Post-test	3.97	0.785		
5	I am interested in some cultural aspects of other countries such as lifestyle, customs and etiquette, art.	Pre-test	3.60	1.117	−3.861	0.000
		Post-test	4.29	0.710		
6	When there is a misunderstanding in the communication with foreigners, I will reflect on and seek solutions.	Pre-test	3.57	0.884	−2.975	0.005
		Post-test	4.17	0.747		
7	In the exchange of different cultures, I am very convinced of my own culture.	Pre-test	3.63	1.087	−2.576	0.015
		Post-test	4.20	0.901		
8	In intercultural communication, I will stand on the other side’s perspective and look at issues instead of taking it for granted.	Pre-test	3.74	0.980	−2.140	0.040
		Post-test	4.26	0.817		
9	I can tolerate cultural differences such as different values, eating habits, taboos, etc.	Pre-test	3.57	1.119	−1.528	0.136
		Post-test	3.91	0.981		
10	I want to understand the cultural differences between China and other countries in terms of values and lifestyles.	Pre-test	3.74	1.146	−3.039	0.005
		Post-test	4.34	0.639		
	*Total*	Pre-test	3.65	0.082	−3.284	0.002
		Post-test	4.20	0.145		

In addition, although the participants were curious about foreign cultures, they had poor English proficiency. They were afraid and worried about intercultural communication. For example, when the teacher introduced the learning activity, most participants thought that they could not complete it because of their limited English ability. However, when the activity started, the participants actively participated and reflected to the teacher that they had many examples from local culture that they would like to introduce to foreign partners with the teacher’s help. From this point, it can be seen that in the learning activities, the attitudes of the participants changed. When encountering difficulties, they actively looked for help. In the interview, some students said that when they completed all the learning activities, the participants found that it was not as difficult as they thought. If there are similar learning activities in the future, they would actively participate.

#### Skills of Discovery and Interaction

The participants scored significantly higher on the post-test (total *M* = 4.29; *SD* = 0.139) than on the pre-test (total *M* = 3.48; *SD* = 0.120), *d* = 1.68, *p* < 0.05 (Table 3). The results show that the participants’ skills of discovery and interaction improved significantly. This suggests that intercultural activities affected the participants’ skills of discovery and interaction related to the foreign cultures they come into contact with.

**TABLE 3:** Skills of discovery and interaction dimension of ICC.

**No.**	**Items**		**Mean**	***SD***	***t***	***p***
1	When communicating with foreigners, I will avoid offending them in language and behavior.	Pre-test	3.60	1.117	−4.100	0.000
		Post-test	4.49	0.702		
2	When there is a misunderstanding in intercultural communication, I will consult and explain Chinese culture with each other, and try to make both parties satisfied.	Pre-test	3.43	1.065	−4.784	0.000
		Post-test	4.43	0.502		
3	When it is difficult to communicate in English, I use body language such as gestures or other non-verbal means.	Pre-test	3.49	1.095	−4.170	0.000
		Post-test	4.31	0.867		
4	I avoid the topic of privacy when communicating with people from different cultures for the first time.	Pre-test	3.60	1.035	−3.295	0.002
		Post-test	4.29	0.667		
5	When looking at cultural events in other countries, such as politics, economy, religion, etc., I will not only start from my own culture and national psychology, but look at problems from as many angles as possible.	Pre-test	3.43	0.979	−4.170	0.000
		Post-test	4.26	0.701		
6	In intercultural exchange and learning activities, I will observe the subtle changes of the other party.	Pre-test	3.54	0.919	−2.741	0.010
		Post-test	4.09	0.781		
7	I will try to avoid prejudice and prejudice when communicating with foreigners.	Pre-test	3.26	0.886	−4.518	0.000
		Post-test	4.17	0.891		
	*Total*	Pre-test	3.48	0.120	−4.974	0.000
		Post-test	4.29	0.139		

The participants said that due to the lack of intercultural communication and learning experience, at first, they could only follow the basic norms of their own country to communicate with foreign partners. Therefore, they were not sure whether their behavior or clothing offended each other, so they could only be as polite as possible in the video. The participants said that they were satisfied with their interaction with foreign partners. One of the important reasons is that they could express themselves smoothly in front of the camera and did not need to read the script. This is something Chinese students could not do but had been trying. Chinese participants thought it was insincere to read the manuscript all the time.

#### Skills of Interpreting and Relating

The participants scored significantly higher on the post-test (total *M* = 3.66; *SD* = 0.326) than on the pre-test (total *M* = 3.03; *SD* = 0.351), *d* = 1.55, *p* < 0.05 (Table 4). The results show that the participants’ skills of interpreting and relating improved significantly. This demonstrates that intercultural activities affected the participants’ skills of interpreting and relating to the foreign cultures they came into contact with.

**TABLE 4:** Interpreting and relating dimension of ICC.

**No.**	**Items**		**Mean**	***SD***	***t***	***p***
1	I am familiar with the cultural connotations of common English words.	Pre-test	2.77	0.843	−3.833	0.001
		Post-test	3.51	0.818		
2	I can gain cultural and intercultural communication knowledge from the exchanges with foreigners.	Pre-test	3.43	0.917	−2.756	0.009
		Post-test	4.03	0.857		
3	I can successfully communicate with people from different cultures and fields (such as different gender, age, social status, religion, etc.).	Pre-test	2.89	0.832	−2.532	0.016
		Post-test	3.43	0.979		
	*Total*	Pre-test	3.03	0.326	−3.607	0.001
		Post-test	3.66	0.351		

The participants said that due to the lack of intercultural experience and intercultural knowledge, they did not know the similarities and differences between the two cultures, nor did they know whether the two cultures are related, and how to explain the differences. This makes it easy for participants to have questions about foreign cultures during the exchange process. Therefore, they were willing to compare the cultures of the two sides and investigate the reasons for similarities and differences deeply to resolve the doubts that they had during the communication process. In the process of investigating the reasons, participants used methods such as asking foreign partners, asking teachers, and using the Internet. One example to show their skills of interpreting and relating is the students mentioned that they found that the price of food in Indonesia is lower than that in China because a Padang rice with rich side dishes only costs 5–10 RMB. It is impossible for a meal of the same specification in China to reach this price. After learning and discussion, participants believe that this may be because Indonesia’s economic development level is not as high as that of China and because Indonesia is located in the tropics, with abundant crops and balanced supply and demand, so food prices are lower than those in China.

#### Critical Cultural Awareness

The participants scored significantly higher on the post-test (total *M* = 4.23; *SD* = 0.181) than on the pre-test (total *M* = 3.59; *SD* = 0.139), *d* = 1.59, *p* < 0.05 (Table 5). The results show that the participants’ critical intercultural awareness was greatly improved. This suggests that intercultural activities have affected participants’ objective understanding of the foreign cultures they come into contact with.

**TABLE 5:** Awareness dimension of ICC.

**No.**	**Items**		**Mean**	***SD***	***t***	***p***
1	When communicating with people from different cultures, I can realize the cultural differences between each other.	Pre-test	3.49	1.173	−3.602	0.001
		Post-test	4.31	0.900		
2	I know my cultural identity in intercultural communication.	Pre-test	3.69	1.078	−3.834	0.001
		Post-test	4.46	0.817		
3	I must have intercultural communication skills in different cultural exchanges.	Pre-test	3.43	0.979	−3.460	0.001
		Post-test	4.23	0.843		
4	When I communicate with foreigners, I know that I will also be restricted by my own culture.	Pre-test	3.43	1.008	−2.416	0.021
		Post-test	4.00	0.804		
5	I can objectively evaluate the behavior of foreigners.	Pre-test	3.49	1.011	−1.365	0.181
		Post-test	3.86	1.033		
6	When communicating with people from different cultures, I can be aware of potential cultural conflicts and try to avoid them.	Pre-test	3.51	0.981	−3.431	0.002
		Post-test	4.20	0.584		
7	I have an objective attitude toward different cultures.	Pre-test	3.66	1.056	−2.953	0.006
		Post-test	4.23	0.598		
8	I think it is necessary to learn different cultural knowledge related to the major.	Pre-test	3.71	1.126	−3.380	0.002
		Post-test	4.43	0.698		
9	I understand that my values will influence me to resolve cultural conflicts in communication.	Pre-test	3.69	0.963	−2.487	0.018
		Post-test	4.23	1.003		
10	I understand the influence of language environment on my language and behavior in intercultural communication.	Pre-test	3.83	1.014	−2.505	0.017
		Post-test	4.31	0.832		
	*Total*	Pre-test	3.59	0.139	−3.885	0.000
		Post-test	4.23	0.181		

The participants mentioned that they never participated in such activities before, so their critical intercultural awareness had been developed after our intercultural learning activities. For example, they found that China is not the only country in the world with rich traditional culture. They found that Indonesia is also a country with rich traditions and culture and is very unique. Discovering the similarities and differences between the two cultures and seeking reasonable explanations helped students improve their critical intercultural awareness. One such example is reflected as the students said they found that Indonesian families need to pray before having dinner. They did not think of this because they believed that only Western countries would pray before. However, Indonesia is a religious country, there are many ethnic groups, and there are many unique rules and customs.

However, there was no significant difference between the two items (item 9 in the Attitude dimension and item 5 in the Awareness dimension) on the pre- and post-test. One reason is that the students participating in the course are students majoring in tourism. Their professional courses helped them become bystanders of different cultures and introduce them to others; this encouraged them to tolerate the taboos, habits and so on of each culture, and so they could objectively look at the behavior of people from different cultures. Therefore, these two items were scored high both at the beginning and at the end of the course. Another reason is that some students mentioned that because communication was not face to face or simultaneous, the taboos, habits and behaviors of foreigners from other cultures could not be observed directly.

### Knowledge Sharing Development

According to the paired sample *t* test, there was a significant difference in the KS (*d* = 1.07; *p* < 0.05) of the participants before and after the experiment (see Table 6). At the same time, if we consider different dimensions of KS (see Table 7), the data show that there was no significant difference in organizational communications (*p* > 0.05), but the difference was significant for personal interactions (*p* < 0.05), and practice (*p* < 0.05).

**TABLE 6:** The paired sample *T*-test of knowledge sharing.

	***N***	***M***	***SD***	***t***	***p***
Pre-test	35	3.81	0.664	−2.276	0.029
Post-test	35	4.11	0.589		
					

**TABLE 7:** The paired sample *T*-test of dimensions of knowledge sharing.

	***N***	**Pre-test**		**Post-test**		***t***	***p***
		***M***	***SD***	***M***	***SD***		
Organizational communications	35	3.91	0.659	4.15	0.555	−1.822	0.077
Personal interactions	35	3.75	0.648	4.06	0.659	−2.132	0.040
Practice	35	3.75	0.894	4.15	0.655	−2.451	0.020

#### Organizational Communications

The participants did not score significantly higher on the post-test (total *M* = 4.15; *SD* = 0.555) than on the pre-test (total *M* = 3.91; *SD* = 0.659), *d* = 1.09; *p* > 0.05. This means that participants did not improve significantly in organizational communications. This showed that students’ knowledge-sharing behaviors, such as expressing opinions and ideas, providing experience, and suggesting solutions to problems, did not improve. This is because of the ceiling effect. That is, students’ organizational communications were basically the same prior to this study. The participants had already experience in group interaction and discussion from previous English and other courses. Therefore, when organizational communication was measured, the score was high at the beginning and end of the study.

#### Personal Interaction

The participants scored significantly higher on the post-test (total *M* = 4.06; *SD* = 0.659) than on the pre-test (total *M* = 3.75; *SD* = 0.648), *d* = 1.06; *p* < 0.05. This means that personal interaction between participants improved. We found that in the past, students in vocational school were not motivated to learn actively after class. However, in the intercultural learning activity of the study, the learning tasks that students had to complete had certain difficulties and challenges, which prompted them to arrange private time after class to complete the tasks and increase interaction among students. After class, when students encountered problems, such as problems with speaking in English or using equipment, they sought help from students who were good at this aspect. In the process of asking for help and providing help, students were engaged in the KS process, particularly in personal interaction.

#### Practice

The participants scored significantly higher on the post-test (total *M* = 4.15; *SD* = 0.655) than on the pre-test (total *M* = 3.75; *SD* = 0.894), *d* = 0.96; *p* < 0.05. This means that the group practice ability of participants was significantly improved. Because intercultural learning activities were not limited to the classroom, students had to present the results of the classroom discussions in the form of 360-degree videos and send them to foreign partners. Therefore, the group members had to gather together to complete the recording task after class. To complete tasks together, students talked, practiced, and shared experiences. This provided more opportunities to improve the practice aspect of KS.

We used Pearson’s correlation coefficient method to analyze the correlation between post-test KS and ICC (Table 8). The codes of the variables are as follows: KS01 – organizational communications of knowledge sharing, KS02 – personal interactions of knowledge sharing, KS03 – practice of knowledge sharing, ICC01 – knowledge of intercultural communicative competence, ICC02 – attitudes of intercultural communicative competence, ICC03 – skills of interpreting and relating of intercultural communicative competence, ICC04 – skills of discovery and interaction of intercultural communicative competence, and ICC05 – critical cultural awareness of intercultural communicative competence.

**TABLE 8:** The correlation of knowledge sharing and ICC.

	**KS01**	**KS02**	**KS03**	**KS**	**ICC01**	**ICC02**	**ICC03**	**ICC04**	**ICC05**	**ICC**
KS01	1									
KS02	0.860**	1								
KS03	0.826**	0.845**	1							
KS	0.946**	0.960**	0.933**	1						
ICC01	0.637**	0.742**	0.612**	0.707**	1					
ICC02	0.760**	0.795**	0.779**	0.821**	0.722**	1				
ICC03	0.687**	0.688**	0.651**	0.715**	0.637**	0.606**	1			
ICC04	0.817**	0.712**	0.700**	0.784**	0.538**	0.744**	0.512**	1		
ICC05	0.752**	0.559**	0.542**	0.651**	0.584**	0.715**	0.576**	0.725**	1	
ICC	0.852**	0.823**	0.768**	0.862**	0.847**	0.917**	0.743**	0.822**	0.862**	1

**Correlation is significant at the 0.01 level (2-tailed).

The results showed that there was a significant correlation between the dimensions of KS and ICC, and the correlation strength was above moderate. The role of individual knowledge for the organization is relatively limited, and only after sharing individual knowledge can it be effectively integrated with other knowledge and then innovated. The exchange and flow of knowledge can also effectively create conditions for the mutual complementation of knowledge among the subjects of knowledge sharing. Knowledge sharing is the key way and driving force for knowledge innovation.

### Participants’ Views on 360-Degree Video Technology

According to descriptive statistics, students had a positive attitude toward the learning activities supported by 360-degree video technology (see section “Appendix 1”). In addition, their levels of satisfaction with technology and perceived usefulness of technology were high.

#### Learning Activities

Most students thought that learning activities supported by technology could promote interaction among students. At the same time, it could also encourage students to acquire and share knowledge in the learning process. After experiencing the learning environment supported by technology, most students were also willing to share their experiences with other students. Students had a higher positive attitude toward the learning environment supported by technology (*M* = 4.29). In the interviews, some students thought that the 360-degree video was very real. Although communication was not synchronous, watching the videos felt that the other person was speaking in front of them. This also relieved their tension in interacting with real people to some extent. In addition, some students mentioned that head-mounted displays also made them more focused on video content.

#### Perceived Satisfaction

Regarding satisfaction with learning by technology, most students were very satisfied with technology as a learning tool and its functions. At the same time, they were also very satisfied with the learning content, learning activities and intercultural exchanges supported by technology. Students were highly satisfied with the learning process supported by technology (*M* = 4.50). The students mentioned that they had not been exposed to 360-degree video before, which was new to them and very interesting. In the process of use, for students, the operation was very simple, and technically, there was not much difficulty. Subtitles in videos could also help them understand the learning content.

#### Perceived Usefulness

Regarding the usefulness of technology, most students believe that learning activities supported by technology could help intercultural learning and enhance ICC and KS. In general, students believed that technology was highly useful for intercultural learning (*M* = 4.18). Some students said that they could learn not only from the content explained by foreign partners but also from the surrounding environments of foreign partners, especially when learning cultural knowledge. In addition, some students said that the bilingual subtitles added to the videos reduced the difficulty of learning in English and enabled them to learn as much as possible about the content in the videos.

However, some students had a lower level of intention to use technology in the future. These students thought that equipment including VR glasses and 360 cameras was too expensive and not very portable. They also said that for individuals, the cost of learning using technology is too high, and it takes considerable money and time. However, if the school or teacher organized a similar unified course again, they will still be willing to participate. In fact, this is the challenge faced by schools and teachers in popularizing courses supported by technology.

### English Proficiency

Before the start of the intercultural project, the English score of the experimental class was *M* = 59.46. After the project, the English score of the students in this class was *M* = 68.93. The paired sample *t*-test showed that there were significant differences between the pre- and post-test, *d* = 0.90; *p* < 0.05 (Table 9). This indicates that in the English classroom with intercultural learning activities, students’ English scores improved significantly.

**TABLE 9:** The paired sample *T*-test of English scores.

	***N***	***M***	***SD***	***t***	***p***
Pre-test	35	59.46	14.510	−6.015	0.000
Post-test	35	68.93	13.441		

## Discussion

This study aimed to answer three research questions. For the first research question, the results showed that intercultural learning activities supported by 360-degree video in the foreign language classroom helped students improve their ICC and ensure their progress in language learning. Compared with previous studies, the conclusion was similar. Chen and Yang (2016) also reported that students’ ICC was mainly developed through the process of intercultural learning so students could acquire cultural knowledge about the target culture. Jung et al. (2019) also found that students’ ICC and language proficiency can be improved through intercultural learning activities. The results of this study support the view that language learning and cultural learning are independent and complementary processes (Damen, 1987). Through analysis, the following conditions may be the reasons for the above conclusion. In this study, intercultural learning activities with foreign peers were learner-centered. Although some students were afraid of difficulties at the beginning of their study, with the help of teachers and other students, they overcame the language barrier. Compared with traditional classrooms, students had a stronger sense of participation in learner-centered classrooms. Second, the cultural topics selected in this study were all related to daily life, which can easily arouse students’ interest and avoid obscure learning content. Finally, the students were participating in an intercultural learning program of this kind for the first time. There was a certain freshness in the course because of learning with foreign partners and using new technology.

Yang (2018) warned that students would feel frustrated when encountering difficulties and seek for assistance in intercultural learning activities because of their limited experience. We paid attention to this issue when designing our learning activity. In the learning process, the teacher and researchers provided timely assistance. Shadiev and Huang (2016) suggested to select topics from students’ daily lives to improve their learning motivation and interests. In his study, we focused on topics suggested by students so for this reason the students showed strong motivation and willingness to participate in the study and communicate with their partners. Therefore, there were favorable conditions in the study to achieve efficient ICC learning.

For the second question, the results showed that, overall, the students’ KS significantly improved in this study. This was consistent with the findings of Wu and Chen (2018) that language learning and group communication with the help of technical tools can enhance students’ KS. However, in terms of the three dimensions of KS, there was no significant difference in student organizational communication. According to interviews and observations, most of the KS in this study took place in group discussions. Students already had prior learning experience in group learning, and their organizational communication was at a high level even before the study. So our activities did not improve this dimension. The following reasons can be drawn. The group formed in this research was too large (7–8 members), so it was not guaranteed that every student could actively participate in group activities, especially with limited class time. Second, in this study, the group activities performed by students were common and lacked any formal evaluation process. That is, the teacher announced a group task and discussion time, and then the students conducted group discussions and obtained certain results, but the teacher did not formally evaluate and gave feedback on the results of the group discussion. According to Bartol and Srivastava (2002), if teachers can provide different levels of rewards or incentives (individual rewards or rewards based on team performance), it may enhance the sharing of personal knowledge within or between groups.

At same time, compared to related studies, the group learning of this research extended the student’s study time beyond the classroom, and it was not limited to the group discussion in the classroom. Therefore, the personal interaction of students has been improved. Moreover, this research designed group tasks that needed to be completed outside the class, so group members also needed to gather together outside of class, which provided opportunities for practice.

In addition, we have another important discovery. The students’ ICC and KS were highly correlated with each other. This showed that KS in intercultural learning and language learning is of great significance. Because sharing increases the value and flow of knowledge, knowledge was further increased (Huysman and Wit, 2004; Nousala and Miles, 2009). Therefore, if members have a higher willingness to share knowledge, greater knowledge benefits will be generated (Bock et al., 2005). The role of individual knowledge for the organization was relatively limited, and only after sharing individual knowledge can it be effectively integrated with other knowledge. The above advantages can enhance not only students’ intercultural knowledge and awareness but also students’ interpreting and relating skills and discovery and interaction skills when sharing knowledge.

For the third research question, the results showed that most students held a positive attitude toward the learning activities supported by 360-degree video technology, were satisfied with the functions of technology, and believed that technology was very useful for improving ICC and promoting KS. The reason is that 360-degree video technology brings a strong sense of reality and immersion. In addition, the tools were easy to operate, very novel and interesting. In the video, in addition to listening to the explanation, students could also obtain more details from the panoramic view. Bilingual subtitles also reduced the difficulty of learning.

Another benefit of using VR in class was the interactive experience students gained through VR to “grasp, retain, and diffuse the gained knowledge amongst others” (Alfalah, 2018), a phenomenon which they would not be able to experience merely through textbooks. Yeh et al. (2020) research also showed how the VR content-creating project enhanced students’ intercultural learning. The students thought that the immersion, one distinct affordance of 360-degree video technology, allowed them to: (1) make sense of and scaffold their prior knowledge with new information of intercultural learning; and (2) organize and structure what they know about their local culture and actively generate the knowledge they have gained into practice (through VR content making and watching); and (3) foster them to be intercultural communicators.

However, some students said they may not use technology (including HMD and 360-degree cameras) in the future, as it is too expensive and less portable. However, if the school organizes related learning activities, they will be more than willing to participate.

## Conclusion

The results of this study showed that in formal foreign language classrooms, intercultural learning supported by 360-degree videos in groups could help students improve their ICC and KS and that ICC and KS can promote each other. Second, it was found that students were satisfied with the learning experience supported by 360-degree video technology. Intercultural learning activities were beneficial for improving students’ ICC, and the process of completing tasks together promoted students’ KS.

The first contribution of this research was that intercultural learning was integrated into formal foreign language classrooms, and real foreign partners were invited to have intercultural exchanges with Chinese students. This was rare in Chinese secondary vocational school classrooms. Second, this research paid attention to the knowledge-sharing behavior of students in intercultural learning. Group learning inside and outside the classroom was conducive to improving the KS of students. Knowledge can create greater value through sharing. Finally, this research also explored the use of 360-degree videos for intercultural learning in formal classrooms.

Intercultural learning activities based on the Byram model showed ways to improve students’ ICC. ICC is an indispensable ability that needs to be cultivated in foreign language education. However, the existing classrooms pay less attention to intercultural learning and rarely carry out targeted intercultural learning activities. Teachers also lack experience in designing intercultural learning activities. At this time, it is necessary to seek a reliable, experienced basis to guide the design of learning activities. Byram’s model is one of the more widely used theoretical models in the field of intercultural teaching. It has high practicability and is very useful for teachers.

This research also promoted the improvement of students’ KS through the process of completing tasks together in groups. This can provide some reference for educators. In addition to the group discussion tasks in the classroom, the classroom tasks can be extended to the after-class period. Communication within the student group provided more opportunities and was more conducive to the improvement of student KS. At the same time, through reflection, the researchers also found that from the perspective of KS, ordinary group learning activities may no longer meet the needs of students. When designing group learning activities, measures such as evaluation activities should be taken to pay attention to the organizational communication within the group, especially for multimember groups.

Compared with other related studies, this research provided a noveler and convenient approach to carry out cross-cultural learning activity supported by 360-degree video technology in formal classrooms. The subtitles in 360 videos also reduced the difficulty of intercultural communication, allowing learners to focus more on cultural content and avoid language barriers. This indicated that educators should provide certain auxiliary measures in learner-centered learning activities. This also reminded researchers that when designing learner-centered learning activities, they need to fully consider the learning ability of learners, imagine the difficulties that students may encounter in learning, and prepare solutions in advance.

## Limitations

This research also had some limitations during the course of its development. Due to availability, Indonesian and Chinese participation formed a one-to-multiple unbalanced partnership. Second, the use of controlled experiments would make the conclusions more convincing. In addition, the subtitles on videos could inhibit the authenticity of contexts recorded by students to some degree. Therefore, we need to find more mature subtitle technologies in the future. Finally, it is worth mentioning that HMD can make learners more focused, but videos that are too long can easily cause user fatigue. When using 360-degree video technology, researchers or teachers need to pay attention to the length of the video.

## Data Availability Statement

The raw data supporting the conclusions of this article will be made available by the authors on reasonable request.

## Ethics Statement

The studies involving human participants were reviewed and approved by School of Education Science, Nanjing Normal University. Written informed consent to participate in this study was provided by the participants’ legal guardian/next of kin.

## Author Contributions

RS contributed to the conception, supervised the research, and was responsible for correspondence. RS, JY, and WS designed the work, collected the data, and analyzed and interpreted data. JY and WS drafted the work and RS substantively revised it. All authors approved the submitted version and agreed both to be personally accountable for the author’s own contributions and to the accuracy the work.

## Conflict of Interest

The authors declare that the research was conducted in the absence of any commercial or financial relationships that could be construed as a potential conflict of interest.

## Publisher’s Note

All claims expressed in this article are solely those of the authors and do not necessarily represent those of their affiliated organizations, or those of the publisher, the editors and the reviewers. Any product that may be evaluated in this article, or claim that may be made by its manufacturer, is not guaranteed or endorsed by the publisher.

## Appendix 1

**TABLE A1:** Usefulness of technology.

**No**	**Dimensions**	**CD^1^**	**AD**	**LA**	**AA**	**CA**	**Mean**	***SD***
	**Learning environments**							
1	I would like to share my learning experience.	1/2.9^2^	0/0	8/22.8	14/40	12/34.3	4.03	0.923
2	I believe technology can assist teacher–learner interaction.	0/0	0/0	7/20	11/31.4	17/48.6	4.29	0.789
3	I believe technology can assist learner–learner interaction.	0/0	0/0	6/17.1	10/28.6	19/54.3	4.37	0.770
4	My classmates would like to share their learning experiences.	0/0	1/2.9	5/14.3	12/34.3	17/48.6	4.29	0.825
5	The technology functions are easy to use to share my cultural knowledge.	0/0	0/0	5/14.3	13/37.1	17/48.6	4.34	0.725
6	The technology functions can improve my cultural knowledge sharing.	0/0	0/0	3/8.6	14/40	18/51.4	4.43	0.655
	*Total*						4.29	
	**Perceived satisfaction**							
7	I am satisfied with using technology as a learning assisted tool.	0/0	0/0	6/17.1	11/31.4	18/51.4	4.34	0.765
8	I am satisfied with using technology functions.	0/0	0/0	1/2.9	14/40	20/57.1	4.54	0.561
9	I am satisfied with learning contents.	0/0	0/0	3/8.6	14/40	18/51.4	4.43	0.655
10	I am satisfied with learning activities.	0/0	0/0		13/37.1	22/62.9	4.63	0.490
11	I am satisfied with intercultural exchange through technology.	0/0	0/0	4/11.4	8/22.9	23/65.7	4.54	0.701
	*Total*						4.50	
	**Perceived usefulness**							
12	I believe technology is useful for ICC.	0/0	0/0	4/11.4	16/45.7	15/42.9	4.31	0.676
13	I believe technology is useful for KS.	0/0	0/0	5/14.3	17/48.6	13/37.1	4.23	0.690
14	I believe using technology is effective for cultural learning.	0/0	0/0	4/11.4	12/34.3	19/54.3	4.43	0.698
15	I believe learning contents are informative.	0/0	0/0	3/8.6	11/31.4	21/60	4.51	0.658
16	I intend to use technology to assist my learning in the future.	1/2.8	2/5.7	12/34.3	10/28.6	10/28.6	3.74	1.039
17	I intend to use technology to assist my learning.	0/0	3/8.6	9/25.7	14/40	9/25.7	3.83	0.923
18	I intend to use technology to improve my learning motivation.	0/0	0/0	9/25.7	10/28.6	16/45.7	4.20	0.833
	*Total*						4.18	

*^1^CD, completely disagree; AD, almost disagree; LA, a little bit agree; AA, almost agree; and CA, completely agree.*

*^2^Number of participants/percentage.*
